# Whey protein mouth drying influenced by thermal denaturation

**DOI:** 10.1016/j.foodqual.2016.03.008

**Published:** 2017-03

**Authors:** Stephanie P. Bull, Yuchun Hong, Vitaliy V. Khutoryanskiy, Jane K. Parker, Marianthi Faka, Lisa Methven

**Affiliations:** a Department of Food and Nutritional Sciences, University of Reading, Whiteknights, Reading, Berks RG6 6AD, United Kingdom; b Department of Pharmacy, University of Reading, Whiteknights, Reading, Berks RG6 6AD, United Kingdom; c Volac International Ltd, 50 Fishers Lane, Orwell, Royston, Hertfordshire SG8 5QX, United Kingdom

**Keywords:** β-LG, β-lactoglobulin, ANOVA, analysis of variance, DLS, dynamic light scattering, QDA, quantitative descriptive analysis, RM-ANOVA, repeated measures analysis of variance, WPC, whey protein concentrate, Whey protein, Drying, Mucoadhesion, Denaturation, Sequential profiling, Particle size

## Abstract

Whey proteins are becoming an increasingly popular functional food ingredient. There are, however, sensory properties associated with whey protein beverages that may hinder the consumption of quantities sufficient to gain the desired nutritional benefits. One such property is mouth drying. The influence of protein structure on the mouthfeel properties of milk proteins has been previously reported. This paper investigates the effect of thermal denaturation of whey proteins on physicochemical properties (viscosity, particle size, zeta-potential, pH), and relates this to the observed sensory properties measured by qualitative descriptive analysis and sequential profiling. Mouthcoating, drying and chalky attributes built up over repeated consumption, with higher intensities for samples subjected to longer heating times (*p* < 0.05). Viscosity, pH, and zeta-potential were found to be similar for all samples, however particle size increased with longer heating times. As the pH of all samples was close to neutral, this implies that neither the precipitation of whey proteins at low pH, nor their acidity, as reported in previous literature, can be the drying mechanisms in this case. The increase in mouth drying with increased heating time suggests that protein denaturation is a contributing factor and a possible mucoadhesive mechanism is discussed.

## 1 Introduction

Whey proteins are becoming an increasingly popular functional food, due to associated health benefits such as the provision of amino acids essential for muscle synthesis (Norton, Wilson, Layman, Moulton, & Garlick, 2012). Recently, whey proteins have been widely utilised in sports nutrition (Wolfe, 2000), the prevention of sarcopenia in elderly and malnourished patients (Dangin et al., 2003), and in a newly developing market for general health and lifestyle products (Chungchunlam et al., 2014, Fekete et al., 2013). The successful use of whey proteins as an aid to muscle growth depends on a consistent intake over an extended period of time (Rahemtulla et al., 2005); therefore the sensory properties of whey protein beverages are of significant importance to ensure a sufficient consumption of protein is achieved. Studies have shown that the mouthfeel of whey protein beverages contributes to the disliking and, therefore, refusal of whey protein beverages, with textural properties being the main reason for 19% of trial discontinuations (of the 56% who completed the questionnaire) (Gosney, 2003). In order to reduce this figure, the mouthfeel properties responsible must be addressed. One major textural aspect of whey proteins is astringency, which was described as a ‘textural defect’ of dairy products in a 1994 review (Lemieux & Simard, 1994). The use of the terms *drying* and *astringency* are often seen as interchangeable, however astringency can often be used to cover a range of different mouthfeel sensations (Gawel, Oberholster, & Francis, 2000), or to specifically refer to “the complex of sensations due to shrinking, drawing or puckering of the epithelium as a result of exposure to substances such as alums or tannins” (ASTM, 2004), which are not present in whey proteins. In this paper the observed sensation of the drying of the mouth will simply be referred to as drying, whereas astringency refers specifically to the puckering of the cheeks.

The nature of the drying sensation elicited by whey proteins is currently unknown, although there have been mechanisms proposed in the literature. As many commercially available whey protein beverages are low pH, the inherent astringency of acidity not the whey proteins themselves, was suggested as the origin of whey protein beverage drying (Lee & Vickers, 2008). An alternative theory is that the interactions between positively charged whey proteins at low pH and negatively charged saliva proteins causes whey protein beverage drying. This can explain the observed correlation between the lowering the pH of whey protein solutions and an increase in both turbidity and drying (Beecher, Drake, Luck, & Foegeding, 2008), and the observation that low pH whey protein beverages are more drying than equivalent pH buffer solutions (Vardhanabhuti, Kelly, Luck, Drake, & Foegeding, 2010). A more recent study elaborates on this theory by proposing that the contribution of salivary proteins to whey protein aggregates at low pH in the mouth reduces the amount of salivary proteins available for oral lubrication; this therefore creates the drying sensation (Ye, Streicher, & Singh, 2011). A variation of this theory proposes the disruption of salivary structure as the cause for astringency in whey protein (Vardhanabhuti, Cox, Norton, & Foegeding, 2011).

Another proposed mechanism is linked to the binding of whey proteins to the oral mucosa (Celebioglu et al., 2015). A study supporting this mechanism found that two whey proteins, β-lactoglobulin (β-LG) and lactoferrin, bound to human oral epithelial cells (Ye, Zheng, Ye, & Singh, 2012). An in vitro study, measuring the binding of two milk proteins, β-LG and casein, to porcine mucosa using fluorescence microscopy, attributed the drying sensation to mucoadhesion (Withers, Cook, Methven, Gosney, & Khutoryanskiy, 2013). Mucoadhesion is the adherence of materials to mucosal membranes, which in this context is the binding of whey proteins to the oral mucosa: the cheeks, gums and tongue. Mucoadhesion occurs via intermolecular forces (electrostatic attraction, hydrophobic interactions and hydrogen bonding) and some covalent bonding such as disulphide bond formation (Andrews et al., 2009, Smart, 2005, Sosnik et al., 2014). The unfolding of whey proteins during denaturation exposes hydrophobic regions and thiol groups (Iametti, DeGregori, Vecchio, & Bonomi, 1996), which could therefore increase the strength of mucoadhesive binding.

As whey proteins are heated they undergo thermal denaturation. This occurs at various temperatures due to the structural differences between the individual proteins in whey. The most abundant protein in bovine whey, β-LG, has a critical temperature of denaturation of 70 °C (Dewit & Swinkels, 1980), with aggregation occurring when temperatures over 70 °C are sustained (Iametti et al., 1996). The denaturation of whey proteins has previously been linked to astringency (Josephson, Thomas, Morr, & Coulter, 1967); this may result from increased hydrophobic interactions or disulphide bonds with the oral mucosae which increase mucoadhesion, finally resulting in increased drying sensation (Hsein, Garrait, Beyssac, & Hoffart, 2015). We hypothesise that particle size will increase upon denaturation due to aggregation, that this will not affect particle charge, but that it will have an effect upon the sensory perception of the sample.

The present study aimed to explore the relationship between denaturation of whey proteins and sensory attributes related to mouth drying. The build-up of sensory properties was analysed using sequential profiling as an indication of potential mucoadhesion.

## 2 Materials and methods

The whey protein concentrate (WPC) used was Volactive Ultrawhey 80 Instant (Volac International Limited, Orwell, Royston, UK), a dry powder with a protein content of 80% minimum, and containing soy lecithin (0.5% maximum) as an emulsifying agent. The remaining 20% contains moisture, fat, lactose, and minerals. Unsalted crackers (Carr’s, United Biscuits, London, UK) were used as palate cleansers in sensory profiling.

### 2.1 Preparation of whey protein beverages

WPC beverages were prepared by addition of WPC powder to water (10% w/v, deionised water). The dilution selected is recommended for many commercially available powders, and represents a serving of 20 g of protein per 250 mL portion, which has been linked to nutritional benefits (Tipton et al., 2007). All samples were stirred for 30 min at room temperature (25 ± 2 °C). A native sample was then stirred for a further 60 min at room temperature (WPC00). Three samples were stirred while being heated in a water bath set at 70 °C for 5, 10 and 20 min (WPC05, WPC10, and WPC20 respectively). A heating temperature of 70 °C was selected as the critical temperature of denaturation for β-LG, the most abundant whey protein (Dewit & Swinkels, 1980). The samples were cooled in a water bath then allowed to hydrate overnight at 4 °C. The pH of all samples ranged from 6.5 to 6.7 (Mettler Toledo SevenEasy, Switzerland; 22 ± 3 °C) and absorbance of light (680 nm; diluted 50 times in water) was measured to quantify sample opacity (Table 1). Measurements were performed in triplicate on each of three processing replicates prepared on three separate days.

### 2.2 Instrumental analysis methods

All instrumental measurements were performed in triplicate on each of three processing replicates prepared on three separate days.

#### 2.2.1 Rheology

Rheological properties of WPC samples were analysed using an oscillatory rheometer (AR2000, TA Instruments, USA) fitted with a 40 mm diameter rotating plate adjusted to 37 °C. Samples were placed on the lower plate surface and equilibrated to 37 °C. Strain sweeps of the samples were obtained by applying oscillation at a frequency of 2 Hz for strain values ranging from 0.01 to 10 in 12 steps. A strain of 1% was then chosen in the linear viscoelastic region for a frequency sweep, where the frequency was varied from 0.1 to 10 Hz in 25 steps.

#### 2.2.2 Dynamic light scattering

WPC samples were diluted 100 times in water (HPLC grade water) for dynamic light scattering (DLS) analysis and measurements were performed using Nano-S Zetasizer (Malvern Instruments, UK) at 30 °C, with an equilibration time of 60 s.

To determine whether any sample sedimentation occurred during the time taken to perform a sensory evaluation, WPC samples were left to stand for 1 h, and then the upper 1 mL and lower 1 mL were assessed using the DLS technique described above.

#### 2.2.3 Zeta-potential

WPC samples were diluted 100 times in water (HPLC grade water) for ζ-potential measurements, which were performed using Nano-S Zetasizer (Malvern Instruments, UK) at 30 °C with an equilibration time of 60 s.

### 2.3 Sensory methods

A trained sensory panel of experts in profiling techniques (*n* = 11; 10 female, 1 male), with a minimum of 6 months training, were given further training on WPC profiling and sequential profiling (minimum 5 h). Sensory evaluation was carried out at room temperature (25 ± 2 °C) in isolated booths.

#### 2.3.1 Quantitative descriptive analysis

Quantitative descriptive analysis (QDA) (Stone, Sidel, Oliver, Woolsey, & Singleton, 1974) was performed using a consensus vocabulary developed by the panel during training (34 attributes; 3 appearance, 6 odour, 6 taste, 6 flavour, 6 mouthfeel, 6 aftereffects, Table A1). The panel assigned mouthfeel characteristics in order to separate important attributes describing distinct sensations. These consensus mouthfeel attributes were: body; furring, the roughening of the tongue; chalky, to describe the sensation of particulate matter; mouthcoating; astringency, specific to the puckering of the cheeks; and drying, to describe the sensation of the reduction of saliva in the mouth.

WPC samples were evaluated in duplicate according to a balanced design using unstructured line scales with appropriate anchors. Samples were presented monadically in opaque white cups (20 mL), unsalted crackers and warm filtered tap water were provided as palate cleansers between samples during an enforced break (2 min). Evaluation was carried out under artificial daylight.

#### 2.3.2 Sequential profiling

Sequential profiling was carried out to establish the perception of seven sensory attributes over repeated consumption of eight aliquots (5 mL) of samples, with 1-min breaks between aliquots. Samples were scored after consumption of each aliquot (T0), and following 30 (T30) and 60 s (T60) time delays, as described by Methven et al. (2010) (Compusense at-hand, Ontario, Canada). Thus there are eight aliquots tasted for each of four samples (WPC00, WPC05, WPC10 and WPC20), scored at three time points (T0, T30 and T60).

The seven attributes scored were bitter, sour, metallic, cooked milk flavour, mouthcoating, chalky and drying. The maximum number of attributes that we recommend to score within one sequential profiling session is 7, determined through training with the panel. These were chosen carefully from the full QDA profile. Bitter, sour and metallic are taste attributes associated with whey protein beverages (Martini and Walsh, 2012, Whetstine et al., 2005). Cooked milk flavour was selected as this attribute showed significant differences between samples in the QDA data as both an odour and flavour attribute. Mouthcoating, chalky and drying were selected by the panel as dominant mouthfeel attributes, and the QDA data showed increases upon heating for all three attributes.

Samples were coded with three-digit numbers and all eight aliquots of one sample were presented together with the same code; the panellists were not blinded to the sequential nature of the evaluation. Warm filtered tap water and unsalted crackers were provided as palate cleansers in the 2 min enforced break between samples; however panellists were instructed not to use these between the eight aliquots of the same sample. Panellists were instructed to consume the total volume of each aliquot and to coat the mouth with the sample before swallowing. Two samples were scored in each session. Evaluation was carried out under red lighting and aliquots were served in opaque black cups to mask appearance differences between samples. Nine of the trained panellists were present for sequential profiling.

### 2.4 Statistical analysis

SENPAQ (version 5.01) was used to carry out analysis of variance (ANOVA) of QDA data. IBM SPSS Statistics (version 21) was used to carry out three-way repeated measures ANOVA (RM-ANOVA) on the sequential profiling data using sample (*n* = 4), assessors (*n* = 9) and repeated consumption (*n* = 8) as explanatory variables. Analytical data were analysed by one-way ANOVA using IBM SPSS Statistics (version 21).

## 3 Results

### 3.1 Instrumental analysis

#### 3.1.1 Rheology

**Fig. 1:**
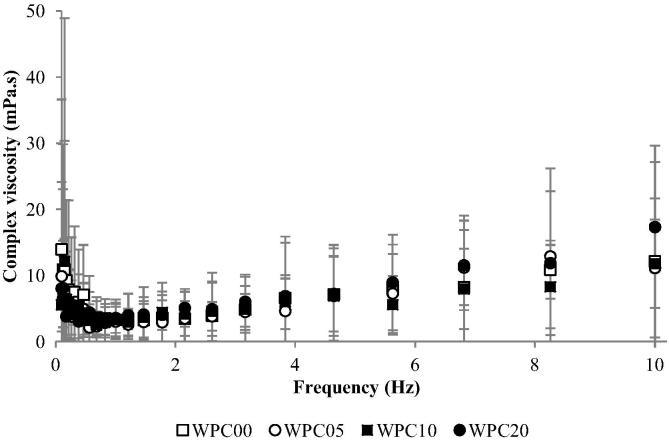
A frequency sweep at a strain of 1% for WPC samples, showing rheological behaviour across a frequency range of 0.1–10 Hz. Error bars represent ±2 standard deviations.

WPC samples were found to have similar viscosities at frequencies between 0.1 and 10 Hz (Fig. 1), showing no significant difference between them (*p* > 0.05).

#### 3.1.2 Dynamic light scattering and ζ-potential measurements

A general increase in particle size diameter (z-average) with an increase in heating time was observed, significant differences were found between all samples, with the exception of WPC05 and WPC10 (*p* ⩽ 0.05). No significant difference was found between Z-average values of the upper and lower 1 mL of sensory samples (taken from a 5 mL sample). Sample charges were determined to be negative for all WPC samples, with no significant difference between ζ-potential magnitudes (*p* > 0.05). These findings are summarised in Table 2.

### 3.2 Sensory data

#### 3.2.1 QDA data

**Fig. 2:**
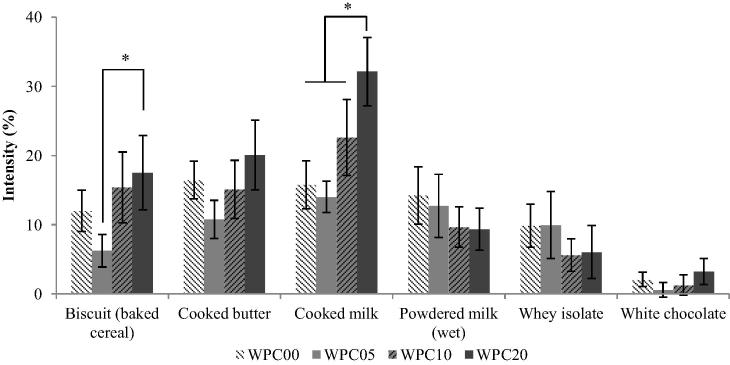
QDA intensities for odour attributes for WPC samples. Error bars represent ±2 standard error of the mean. ^*^ Significantly different scores between samples (*p* < 0.05) calculated through ANOVA.

**Fig. 3:**
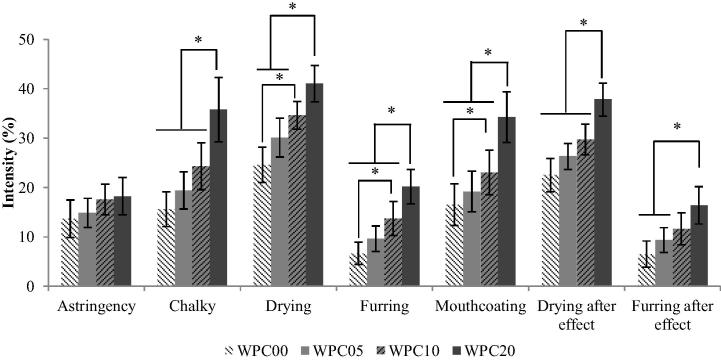
QDA intensities for mouthfeel and aftereffect attributes related to mouth drying for WPC samples. Error bars represent ±2 standard error of the mean. ^*^ Significantly different scores between samples (*p* < 0.05) calculated through ANOVA.

Of the 34 attributes evaluated, 15 were significantly different between samples, as outlined in Table A1. The appearance attributes were important, as any visual differences between samples would require masking for an unbiased evaluation. Significant differences were found for both beige colour and body appearance attributes, therefore red light was a requirement for further evaluation of samples by sequential profiling. Taste attributes showed little or no change across samples with increasing heating times. Across biscuit (baked cereal), cooked butter and cooked milk odour and flavour attributes, WPC00 had a higher intensity score than WPC05, however there was an upward trend in intensity across WPC05 to WPC10 and WPC20 (scores for odour attributes shown in Fig. 2). A significant increase in intensity of mouthfeel attributes was seen for samples with longer heating times, including drying but with the exception of astringency (see Fig. 3).

#### 3.2.2 Sequential profiling data

**Fig. 4:**
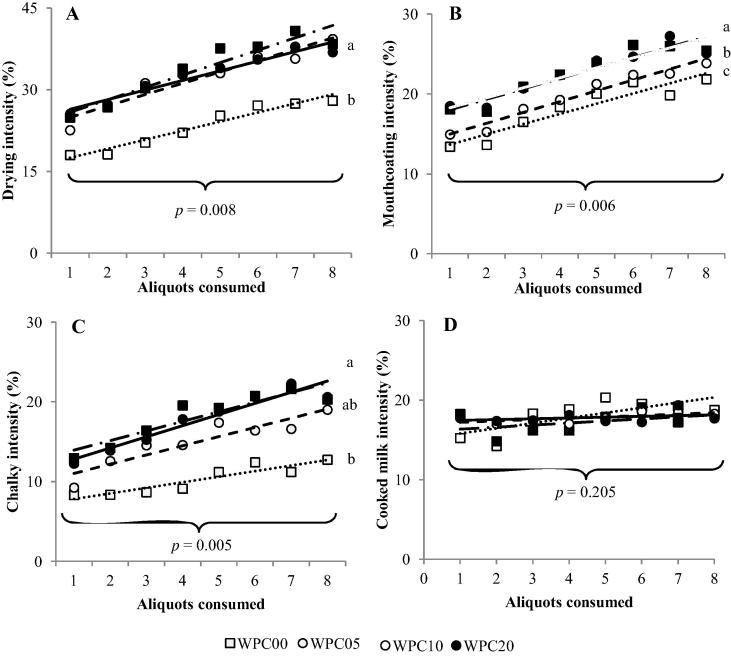
Mean intensities scored during consumption (T0) from sequential profiling of WPC samples over 8 repeated consumptions. Letters denote significantly different sample groupings as calculated by RM-ANOVA; *p*-values are shown for significant changes over repeated consumption. A: drying. Rates of incline: WPC00, 1.7 (R^2^ = 0.951; WPC05, 2.1 (R^2^ = 0.919); WPC10, 2.3 (R^2^ = 0.898); WPC20, 1.8 (R^2^ = 0.942). B: mouthcoating. Rates of incline: WPC00, 1.3 (R^2^ = 0.882); WPC05, 1.4 (R^2^ = 0.959); WPC10, 1.3 (R^2^ = 0.891); WPC20, 1.3 (R^2^ = 0.886). C: chalky. Rates of incline: WPC00, 0.7 (R^2^ = 0.855); WPC05, 1.2 (R^2^ = 0.850); WPC10, 1.2 (R^2^ = 0.844); WPC20, 1.4 (R^2^ = 0.920). D: cooked milk. Rates of incline: WPC00, 0.7 (R^2^ = 0.549); WPC05, 0.3 (R^2^ = 0.204); WPC10, 0.2 (R^2^ = 0.492); WPC20, 0.1 (R^2^ = 0.125).

Data from sequential profiling was collected to observe the change in intensity of attributes over repeat consumption of 40 mL of WPC samples. Significant differences (*p* < 0.05) between WPC samples were found overall for drying, mouthcoating and chalky attributes: WPC00 had a significantly lower drying score than the heated WPC samples; WPC10 and WPC20 were found to have significantly higher mouthcoating intensities than WPC05, which had a significantly higher mouthcoating intensity than WPC00. A general increase in chalky intensity was observed for samples with longer heating times (Fig. 4). These data support those collected by QDA. These attributes were also found to increase significantly with repeated consumption, with rates of incline (Δ intensity/aliquot) ranging from 0.7 (WPC00 chalky T0) to 2.9 (WPC10 drying T60) (average rates of incline shown in Table 3).

In contrast, cooked milk, bitter, sour, and metallic attributes showed no significant differences between samples neither during consumption nor during aftertaste ratings (Table 3). Some significant differences (detail in Table 3) were seen for these attributes across repeated consumption: bitter T0, T30, T60; metallic and sour T30, T60; cooked milk T60. Rates of incline (Δ intensity/aliquot) ranged from −0.2 (WPC00 metallic T0) to 0.7 (WPC20 cooked milk T30) (Table 3).

**Fig. 5:**
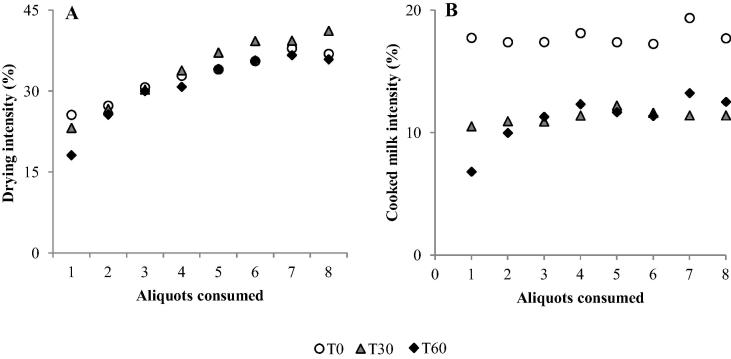
Mean intensities scored for WPC20 showing T0, T30, and T60 as separate data sets for A: drying, where little difference is seen between T0, T30 and T60; and B: cooked milk, where a decrease in intensity is observed for T30 and T60 in comparison to T0.

To quantify the relative strength of the intensity scores for aftertaste results, the mean intensity scores at T30 and T60 were calculated as percentages of the equivalent T0 score (Table 4). This provides a comparison of how much the attribute intensity increased or decreased post consumption. These results showed high aftertaste scores for drying (95–112% intensity compared to the T0 score), mouthcoating (89–104%), chalky (83–101%), and bitter (81–96%); and lower aftertaste scores for metallic (63–91%), sour (64–82%) and cooked milk (56–77%); (drying and cooked milk represented graphically in Fig. 5).

## 4 Discussion

The range of significantly different attributes between samples evaluated by QDA shows that the heat treatment of WPC samples can significantly affect the sensory profile. For example, as little heating as possible would be recommended in order to decrease the amount of mouth drying sensation in a product; whereas heating a sample at 70 °C would be preferable to increase the amount of cooked milk odour in a product. The majority of significant differences were found in appearance, mouthfeel and aftereffect attributes (Table A1).

Odour and flavour attributes which showed significant differences between samples were: biscuit (baked cereal) (odour only); cooked butter (flavour only); and cooked milk (both flavour and odour). These attributes followed a trend of a general increase in intensity with samples that had longer heating times, with the exception of WPC00, which scored higher than WPC05, and in some cases WPC10 or WPC20 (Fig. 2). The proposed reason for this is that upon initial heating, existing volatile flavour molecules are initially lost as the vapour pressure of the sample is increased; therefore a decrease in flavour and odour attributes is seen between WPC00 and WPC05. However upon further heating of the sample, new volatile molecules are created through the release of thiol compounds from denatured proteins, the pyrolysis of sugar, and the degradation of amino acids, among other heat-induced mechanisms (Calvo & de la Hoz, 1992). Further experiments would be required to study the changes in flavour chemistry upon heating.

The differences in appearance attributes for samples are important as these required masking for sequential profiling. As a result, samples were presented monadically in opaque black cups under red lighting. The upper and lower 1 mL of sensory samples were analysed using DLS to ensure that sedimentation was not significant over the time taken to evaluate samples. There was no significant difference found between samples, and therefore no sedimentation effect was likely to have occurred during sensory evaluation.

The samples needed to have similar viscosities in order to control the sensory experiments, as differences in viscosity can affect sensory perception of both mouthfeel and texture attributes (Courregelongue, Schlich, & Noble, 1999), however in previous research by Beecher et al. (2008), viscosity was not found to have an effect on drying. The rheological analysis of WPC samples showed no difference between samples over frequencies of 0.1–10 Hz, corresponding to oral shear rates predicted in the mouth (Cutler et al., 1983, Shama and Sherman, 1973). This is partially reflected by the QDA results for the body mouthfeel attribute: no significant differences were found between samples with the exception of WPC20, which scored higher than the other samples.

The QDA found that all mouthfeel attributes showed a general trend of increasing attribute intensity with samples that had undergone longer heating times, and all mouthfeel attributes except astringency showed some differentiation between samples (Fig. 3; Table A1). We concluded that chalky, drying, furring and mouthcoating all increased upon heating. These attributes were all positively correlated with correlation coefficients of between 0.96 and 1. The sequential profiling results also concluded that chalky, mouthcoating and drying increased with repeated consumption, which could contribute to the drying sensations preventing full consumption of whey protein beverages (Gosney, 2003).

The significant difference found for drying between WPC00 and the three heated samples (WPC05, WPC10 and WPC20) proves the hypothesis that drying increases with heating. This finding also indicates that samples which have been heated for over 5 min at 70 °C have a significantly increased intensity of drying, which could be caused by the denaturation of whey proteins at these conditions.

The sequential profiling results for mouthcoating support a mechanism of whey protein mucoadhesion. Samples showed significant differences for mouthcoating: WPC05 scored significantly higher than WPC00; and WPC10 and WPC20 scored significantly higher than WPC05. These differences indicate that mouthcoating significantly increases before 5 to 10 min of heating at 70 °C, however there is no difference seen for further heating from 10 to 20 min. This increase in mouthcoating could be due to an increase in mucoadhesion caused by the denaturation of protein in the samples (Hsein et al., 2015). The increase of mouthcoating and drying over repeated consumption also supports the mucoadhesion theory, as the observed build-up of these attributes suggests a physical increase of sensation-causing substance in the mouth, which would be consistent with mucoadhesion. The aftertaste intensities (T30 and T60) are high for chalky, mouthcoating and drying, demonstrating that these sensations are just as prominent once the sample has been swallowed. This has been previously reported in whey-rich ingredients by Withers, Lewis, Gosney, and Methven (2014), and could be due to mucoadhesion of the whey proteins.

DLS can be used to measure the average particle size in solution, and to determine the change in particle size upon aggregation of a sample. The aggregation of isolated whey proteins, in particular β-LG, has been well studied using light scattering techniques (Elofsson et al., 1996, Mehalebi et al., 2008); however when heating WPC, which contains a mixture of the different whey proteins, and other constituents such as lactose, fats and minerals, different denaturation and aggregation behaviour is observed. When heated in the presence of other whey proteins β-LG forms both homopolymers and heteropolymers (Havea, Singh, & Creamer, 2001), and in the presence of caseins large micelles can be formed upon aggregation (Havea, 2006). This formation of large particles could explain the large particle sizes observed for the WPC samples, in comparison to those formed by isolated proteins. The z-averages calculated from DLS measurements give an indication to the average particle size in WPC samples, however as the sample is unlikely to be monodisperse due to the range of constituents in the mixture, these values are provided only as a comparative guide to the change observed upon heating of samples. A positive correlation was observed between the heating time and z-average particle size, with significant differences found between samples. This increase is likely to be caused by an increase in the size of aggregates caused by higher levels of denaturation.

The observation that larger particle sizes can contribute to the “astringency” of whey protein has been previously reported by Ye et al. (2011). The increase observed in chalky upon heating could be caused by the increase in particle size with longer heating times, however the increase in chalky over repeated consumption indicates that particle size is not the sole contributor to mouthfeel attributes in the samples, which could be caused by a build-up of these particles by mucoadhesion.

All WPC samples were found to have a negative charge with ζ-potentials of similar magnitude, which is expected due to the similar pH of samples. As the samples have similar ζ-potentials, it is unlikely that the differences in drying perception between WPC samples in this study arise from electrostatic interactions with saliva proteins, as predicted by Ye et al. (2011). Although the electrostatic interactions could still be occurring, the differences between the samples must be caused by another mechanism.

## 5 Conclusions

Whey protein samples were heated for varying times (0, 5, 10 and 20 min; 70 °C) and the pH, viscosity, particle size, and ζ-potential were measured. All WPC samples were found to have similar pH, viscosity and ζ-potentials, indicating that previously proposed mechanisms for whey protein drying based on these properties (Lee and Vickers, 2008, Vardhanabhuti et al., 2010, Ye et al., 2011) cannot explain the changes in drying and related attributes which varied significantly between WPC samples. The z-averages of WPC samples increased with longer heating times, indicative of aggregation caused by the denaturation of a mixture of whey proteins (Havea et al., 2001).

Drying, mouthcoating and chalky attributes were found to increase for samples with longer heating times, with the intensity of these attributes building up with the repeated consumption of sample. These findings are compatible with the proposed mechanism of mucoadhesion as the source of whey drying, supported by previous studies (Withers et al., 2013), and denaturation increasing mucoadhesive strength (Hsein et al., 2015).

Further research is required to determine the mucoadhesive properties of whey proteins. Investigations will be carried out to establish the mechanism of action for the adhesion of whey proteins to the oral mucosa, how this is influenced by protein structure and denaturation, and how a drying sensation is elicited by this mechanism.

## Figures and Tables

**Table A1:** QDA attributes and reference descriptions or standard, with results for WPC samples. Lettering indicates significantly different groupings (*p* < 0.05).

Modality	Attribute	Reference description	WPC00	WPC05	WPC10	WPC20
Appearance	Beige colour	Degree of beige colour intensity	40.2^a^	26.5^b^	21.3^bc^	13.1^c^
Appearance	Body	Fullness of sample	20.5^b^	23.1^ab^	24.5^ab^	27.4^a^
Appearance	Opacity	Overall opacity of sample	82.9	86.5	85.2	89.7
Odour	Biscuit (baked cereal)	Baked cereal element of a digestive biscuit	12.0^ab^	6.3^b^	15.4^ab^	17.5^a^
Odour	Cooked butter	Unsalted butter fully melted	16.5	10.8	15.1	20.1
Odour	Cooked milk	Semi-skimmed milk heated in a microwave for 3 min	15.8^b^	14.0^b^	22.6^b^	32.1^a^
Odour	Powdered milk (wet)	10% skim powdered milk in deionised water	14.2	12.7	9.7	9.4
Odour	Whey isolate	WPI90 (5% in deionised water; Volac)	9.9	10.0	5.6	6.1
Odour	White chocolate	White chocolate (Nestlé, Milkybar)	2.1	0.6	1.3	3.3
Taste	Sour	Citric acid (0.76 g/L)	14.2	14.1	15.3	17.2
Taste	Bitter	Quinine (0.04 g/L)	15.5	13.0	15.1	15.0
Taste	Metallic	Iron (II) sulfate heptahydrate (0.0036 g/L)	14.3	13.1	13.6	11.3
Taste	Salty	Sodium chloride (1.19 g/L)	2.4	1.6	3.4	2.8
Taste	Sweet	Sucrose (5.76 g/L)	5.8^ab^	5.0^b^	6.0^ab^	8.8^a^
Taste	Umami	Monosodium glutamate (0.29 g/L)	2.6	1.4	2.2	2.9
Flavour	Biscuit (baked cereal)	Baked cereal element of a digestive biscuit	11.2	8.0	11.9	11.8
Flavour	Cooked butter	Unsalted butter fully melted	13.4^a^	6.5^b^	8.1^ab^	12.8^ab^
Flavour	Cooked milk	Semi-skimmed milk heated for 3 min	15.6^b^	12.3^b^	19.3^ab^	25.3^a^
Flavour	Powdered milk (wet)	10% skim powdered milk in deionised water	12.7	11.7	11.2	11.2
Flavour	Whey isolate	WPI90 (5% in deionised water; Volac)	8.9	9.0	7.2	3.8
Flavour	White chocolate	White chocolate (Nestlé, Milkybar)	2.0	0.5	1.5	1.8
Mouthfeel	Astringency	Puckering of the cheeks	13.7	14.9	17.6	18.2
Mouthfeel	Body	Fullness of sample	17.2^b^	18.0^b^	18.5^b^	30.2^a^
Mouthfeel	Chalky	Dry fine insoluble powder	15.6^b^	19.4^b^	24.3^b^	35.8^a^
Mouthfeel	Drying	The absorbance of moisture from the mouth	24.6^c^	30.1^bc^	34.6^ab^	41.0^a^
Mouthfeel	Furring	Rough ‘furry’ texture on tongue and mouth	6.7^c^	9.6^bc^	13.8^b^	20.2^a^
Mouthfeel	Mouthcoating	Degree of coating of the mouth	16.5^c^	19.2^bc^	23.0^b^	34.3^a^
Aftereffect	Aftertaste strength	The strength of the overall aftertaste	18.4	17.5	20.7	21.9
Aftereffect	Bitter	Quinine (0.04 g/L)	6.2	6.3	7.1	9.0
Aftereffect	Drying	The absorbance of moisture from the mouth	22.5^b^	26.3^b^	29.7^b^	37.8^a^
Aftereffect	Furring	Rough ‘furry’ texture on tongue and mouth	6.5^b^	9.4^b^	11.6^ab^	16.4^a^
Aftereffect	Metallic	Iron (II) sulfate heptahydrate (0.0036 g/L)	10.7	8.2	7.9	8.2
Aftereffect	Sour	Citric acid (0.76 g/L)	3.9^b^	4.9^ab^	5.7^ab^	7.4^a^

**Table 1:** Heating time, pH, and absorbance of light at 680 nm (samples diluted 50 times in water) for WPC samples. Errors represent ±2 standard deviations.

Sample	Heating time at 70 °C (min)	pH	Absorbance at 680 nm
WPC00	0	6.60 ± 0.04	0.098 ± 0.021
WPC05	5	6.62 ± 0.04	0.149 ± 0.025
WPC10	10	6.63 ± 0.04	0.170 ± 0.023
WPC20	20	6.64 ± 0.03	0.222 ± 0.088

**Table 2:** The z-averages of WPC samples measured from the bulk, upper 1 mL and lower 1 mL of samples allowed to stand, as measured by DLS; and the ζ-potentials of WPC samples. Errors represent ±2 standard deviations.

Sample	Z-average (nm)			ζ-potential (mV)
	Bulk sample	Upper sample	Lower sample	
WPC00	220 ± 16	224 ± 11	219 ± 11	−27.7 ± 3.1
WPC05	272 ± 15	293 ± 15	282 ± 10	−26.7 ± 2.6
WPC10	288 ± 19	299 ± 26	289 ± 24	−27.0 ± 3.9
WPC20	317 ± 71	335 ± 25	321 ± 21	−26.2 ± 4.0

**Table 3:** Significance levels between samples are shown: no significant difference; and a significant difference, *p* < 0.05 (*). Significance levels over repeat consumption for tasting (T0) and aftertaste at 30 (T30) and 60 s (T60), from RM-ANOVA of sequential profiling data. The average rates of incline (Δ intensity/aliquot) are shown beside significance levels.

Attribute	Sample significant differences			Rate of incline over repeat consumption		
	T0	T30	T60	T0	T30	T60
Cooked Milk	ns	ns	ns	ns (0.3)	ns (0.4)	^∗^(0.4)
Sour	ns	ns	ns	ns (0.2)	^∗^(0.5)	^∗^(0.5)
Metallic	ns	ns	ns	ns (0.1)	^∗^(0.2)	^∗^(0.2)
Bitter	ns	ns	ns	^∗^(0.4)	^∗^(0.4)	^∗^(0.5)
Chalky	^∗^	^∗^	^∗^	^∗^(1.1)	^∗^(1.3)	^∗^(1.3)
Drying	^∗^	^∗^	^∗^	^∗^(1.9)	^∗^(2.4)	^∗^(2.4)
Mouthcoating	^∗^	^∗^	^∗^	^∗^(1.3)	^∗^(1.6)	^∗^(1.6)

**Table 4:** Relative strength of aftertaste, expressed as a percentage of the T0 score, for T30 and T60. Values are shown for the eighth aliquot scores for all attributes.

Attribute	T30				T60			
	WPC00	WPC05	WPC10	WPC20	WPC00	WPC05	WPC10	WPC20
Cooked Milk	65%	65%	62%	77%	61%	59%	56%	71%
Sour	73%	82%	80%	65%	71%	64%	70%	59%
Metallic	63%	89%	84%	87%	63%	76%	91%	63%
Bitter	91%	94%	74%	83%	87%	84%	81%	96%
Chalky	98%	92%	101%	95%	94%	89%	97%	83%
Drying	103%	99%	105%	112%	96%	95%	101%	97%
Mouthcoating	92%	104%	99%	101%	89%	99%	95%	96%

## Appendix A

See Table A1.
